# Supplementary material for: A Critical View of the Application of the APEX Software (Aqueous Photochemistry of Environmentally-Occurring Xenobiotics) to Predict Photoreaction Kinetics in Surface Freshwaters
Source: Molecules. 2019 Dec 18;25(1):9. doi: 10.3390/molecules25010009 (PMC7017383; doi:10.3390/molecules25010009)
Supplement: Supplementary file 1 [file molecules-25-00009-s001.zip › 3.2.4_gcc-4.4.0.zip!/3.2.4_gcc-4.4.0/3.2.4_gcc-4.4.0/doc/octave/html/interpreter/Index-Expressions.html]

Index Expressions - Untitled


Next: Calling Functions,
Up: Expressions


---

### 8.1 Index Expressions

An index expression allows you to reference or extract selected
elements of a matrix or vector.

Indices may be scalars, vectors, ranges, or the special operator
:, which may be used to select entire rows or columns.

Vectors are indexed using a single index expression. Matrices may be
indexed using one or two indices. When using a single index
expression, the elements of the matrix are taken in column-first order;
the dimensions of the output match those of the index expression. For
example,

```
     a (2)       # a scalar
     a (1:2)     # a row vector
     a ([1; 2])  # a column vector
```

As a special case, when a colon is used as a single index, the output
is a column vector containing all the elements of the vector or matrix.
For example

```
     a (:)       # a column vector
```

Given the matrix

```
     a = [1, 2; 3, 4]
```

all of the following expressions are equivalent

```
     a (1, [1, 2])
     a (1, 1:2)
     a (1, :)
```

and select the first row of the matrix.

In general, an array with n dimensions can be indexed using m
indices. If `n == m`, each index corresponds to its respective dimension.
The set of index tuples determining the result is formed by the Cartesian product
of the index vectors (or ranges or scalars).
If `n < m`, then the array is padded by trailing singleton dimensions.
If `n > m`, the last `n-m+1` dimensions are folded into a single
dimension with extent equal to product of extents of the original dimensions.

Indexing a scalar with a vector of ones can be used to create a
vector the same size as the index vector, with each element equal to
the value of the original scalar. For example, the following statements

```
     a = 13;
     a (ones (1, 4))
```

produce a vector whose four elements are all equal to 13.

Similarly, indexing a scalar with two vectors of ones can be used to
create a matrix. For example the following statements

```
     a = 13;
     a (ones (1, 2), ones (1, 3))
```

create a 2 by 3 matrix with all elements equal to 13.

The last example could also be written as

```
     13 (ones (2, 3))
```

It should be, noted that `ones (1, n)` (a row vector of ones) results in a
range (with zero increment), and is therefore more efficient when used in index
expression than other forms of ones. In particular, when r is a row
vector, the expressions

```
       r(ones (1, n), :)
```

```
       r(ones (n, 1), :)
```

will produce identical results, but the first one will be significantly
faster, at least for r and n large enough. The reason is that
in the first case the index is kept in a compressed form, which allows Octave
to choose a more efficient algorithm to handle the expression.

In general, for an user unaware of these subtleties, it is best to use
the function repmat for spreading arrays into bigger ones.

It is also possible to create a matrix with different values. The
following example creates a 10 dimensional row vector a containing
the values
a(i) = sqrt(i).

```
     for i = 1:10
       a(i) = sqrt (i);
     endfor
```

Note that it is quite inefficient to create a vector using a loop like
the one shown in the example above. In this particular case, it would
have been much more efficient to use the expression

```
     a = sqrt (1:10);
```

thus avoiding the loop entirely. In cases where a loop is still
required, or a number of values must be combined to form a larger
matrix, it is generally much faster to set the size of the matrix first,
and then insert elements using indexing commands. For example, given a
matrix `a`,

```
     [nr, nc] = size (a);
     x = zeros (nr, n * nc);
     for i = 1:n
       x(:,(i-1)*nc+1:i*nc) = a;
     endfor
```

is considerably faster than

```
     x = a;
     for i = 1:n-1
       x = [x, a];
     endfor
```

particularly for large matrices because Octave does not have to
repeatedly resize the result.

— Function File: ind = **sub2ind** (dims, i, j)  
— Function File: ind = **sub2ind** (dims, s1, s2, ..., sN)  
> Convert subscripts into a linear index.
>
> The following example shows how to convert the two-dimensional
> index `(2,3)` of a 3-by-3 matrix to a linear index. The matrix
> is linearly indexed moving from one column to next, filling up
> all rows in each column.
>
> ```
>           linear_index = sub2ind ([3, 3], 2, 3)
>           => 8
> ```
>
>
>
>
> **See also:** ind2sub.

— Function File: [s1, s2, ..., sN] = **ind2sub** (dims, ind)  
> Convert a linear index into subscripts.
>
> The following example shows how to convert the linear index `8`
> in a 3-by-3 matrix into a subscript. The matrix is linearly indexed
> moving from one column to next, filling up all rows in each column.
>
> ```
>           [r, c] = ind2sub ([3, 3], 8)
>           => r =  2
>           c =  3
> ```
>
>
>
>
> **See also:** sub2ind.
